# Immune Checkpoint Inhibitor–Associated Myocarditis With Multisystem Immune Toxicity in Renal Cell Carcinoma

**DOI:** 10.1016/j.jaccas.2026.107003

**Published:** 2026-02-23

**Authors:** Raj Nandan Chennuri, Makarand Madine, Reshmanth Prathipati, Raam Mannam, Sandhya Rani Reddy, Prudhvi Davala, Jaswinder Kaur Chilana, Fazal Bari

**Affiliations:** a Department of Internal Medicine, NYMC/St. Clare's and St. Mary's Hospital, Denville, New Jersey, USA; b Department of Oncology & Hematology, Oncology & Hematology Specialists Group, Denville, New Jersey, USA

**Keywords:** blood tests, cancer, myocardial ischemia

## Abstract

**Background:**

Immune checkpoint inhibitors are widely used in the adjuvant treatment of renal cell carcinoma. Although immune-related adverse effects are well described, myocarditis remains an uncommon complication. Ealy multisystem immune-related toxicity after limited exposure to therapy is uncommon and may be difficult to recognize in older patients.

**Case Summary:**

An 84-year-old woman receiving adjuvant pembrolizumab for renal cell carcinoma presented with dyspnea shortly after her second dose. She was found to have a new left bundle branch block, with marked troponin elevation and mild left ventricular dysfunction. Additional findings were consistent with myositis, pneumonitis, and nephritis. Coronary angiography showed nonobstructive coronary disease. Pembrolizumab was discontinued, and high-dose corticosteroids were initiated, resulting in gradual clinical improvement.

**Discussion:**

This case shows the early presentation of pembrolizumab-associated myocarditis with multisystem immune-related toxicity involvement in an elderly patient, and it highlights the importance of early recognition and multidisciplinary management.

**Take-Home Message:**

Early recognition and rapid initiation of corticosteroids are critical in suspected immune checkpoint inhibitor–associated myocarditis.

## Graphical Abstract

**Figure:**
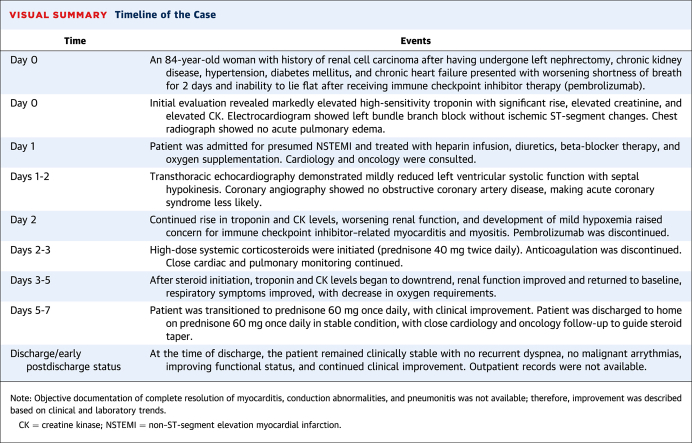

Immune checkpoint inhibitors (ICIs) have expanded into adjuvant therapy for renal cell carcinoma.^1^ While immune-related adverse events are common, ICI-associated myocarditis is rare but potentially severe, and it may present early, sometimes after just 1 or 2 doses.^2^ Concomitant immune toxicities, including myositis, pneumonitis, and nephritis, can occur and may complicate diagnosis, particularly in older patients with baseline cardiovascular and renal comorbidities. We describe an elderly patient receiving adjuvant pembrolizumab who developed early myocarditis with multisystem immune toxicity requiring rapid multidisciplinary evaluation and treatment.

**Take-Home Messages**

• Immune checkpoint inhibitor–associated myocarditis can present early and involve multiple organ systems, particularly in older patients with baseline comorbidities.
• Early recognition and initiation of high-dose corticosteroids are critical to prevent progression to severe or fulminant disease.

## History of Presentation

An 84-year-old woman with renal cell carcinoma presented to the emergency department with worsening shortness of breath for 2 days and inability to lie flat. She denied chest pain, palpitations, syncope, fever, or recent viral symptoms. She had recently received adjuvant pembrolizumab for renal cell carcinoma, and her symptoms developed shortly after receiving the second dose.

## Past Medical History

The patient's medical history was notable for renal cell carcinoma (pathologic stage pT3) after having undergone left radical nephrectomy, chronic kidney disease stage 3 (baseline creatinine approximately 2 mg/dL), hypertension, hyperlipidemia, diabetes mellitus, diastolic heart failure, and known coronary artery disease without prior myocardial infarction.

## Differential Diagnosis

The initial differential diagnosis included acute coronary syndrome, pulmonary embolism, stress-induced cardiomyopathy, viral myocarditis, decompensated heart failure, pneumonia, and ICI-associated myocarditis with multisystem immune-related toxicity.

## Investigations

**Figure 1:**
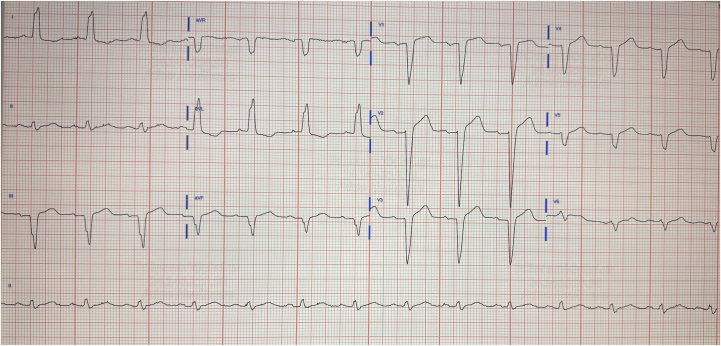
Electrocardiogram Demonstrating New Left Bundle Branch Block After Pembrolizumab Initiation

On presentation, the patient was afebrile with heart rate 81 beats/min, respiratory rate 21 breaths/min, blood pressure 141/74 mm Hg, and oxygen saturation of 97% on 3 L nasal cannula. Electrocardiogram demonstrated a new left bundle branch block (LBBB) (Figure 1). Laboratory evaluation revealed markedly elevated high-sensitivity troponin of 4,497 ng/L, later peaking at 6,598 ng/L, with B-type natriuretic peptide 96.1 pg/mL and D-dimer 0.78 (age-adjusted low). Creatine kinase (CK) was elevated to 4,147 U/L, consistent with myositis and rhabdomyolysis. Creatinine was 2.89 mg/dL (above baseline). Liver function tests were within normal limits. Complete blood count did not reveal leukocytosis or cytopenias. Urinalysis revealed proteinuria and hematuria without evidence of infection. Respiratory viral panel, rapid influenza A/B, and SARS-CoV-2 polymerase chain reaction were negative. Erythrocyte sedimentation rate, C-reactive protein, and other viral titers were not obtained during hospitalization.

Baseline electrocardiogram and transthoracic echocardiogram immediately prior to initiation of ICI therapy were not available for review; the most recent cardiac testing had been performed approximately 5 months earlier and reportedly demonstrated preserved left ventricular systolic function (left ventricular ejection fraction >55%), with sinus rhythm on electrocardiogram without documented conduction abnormalities. Transthoracic echocardiography during the current admission revealed mildly reduced left ventricular systolic function with an estimated ejection fraction of 45% to 50% and septal hypokinesis. Global longitudinal strain was not assessed.

**Figure 2:**
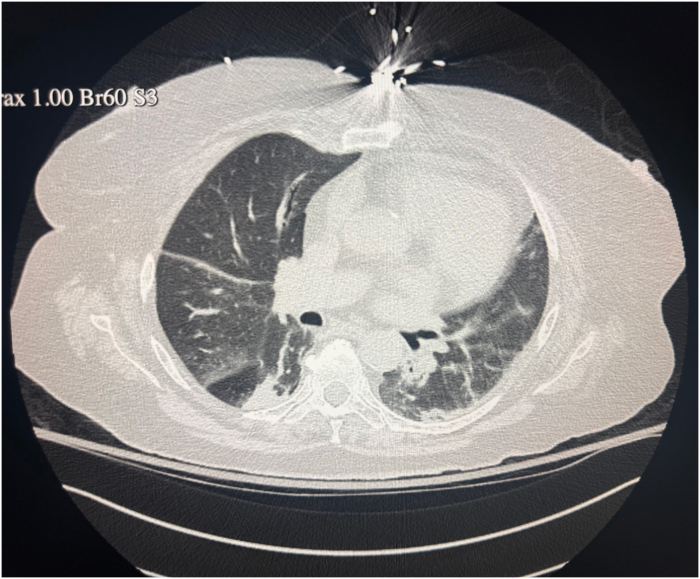
Chest Computed Tomography Demonstrating Bilateral Ground-Glass Opacities Concerning for Immune Checkpoint Inhibitor–Associated Pneumonitis

Coronary angiography demonstrated nonobstructive coronary artery disease. High-resolution computed tomography of the chest demonstrated bilateral basilar consolidations and ground-glass opacities, concerning for immune-related pneumonitis (Figure 2). Infectious evaluation was negative.

There were no clinical features suggestive of myasthenia gravis, including ptosis, diplopia, bulbar symptoms, or proximal muscle weakness; acetylcholine receptor antibody testing was not performed owing to low clinical suspicion.

## Management

Given the new LBBB and markedly elevated troponin, the patient was initially treated for possible non–ST-segment elevation myocardial infarction with heparin infusion and close monitoring. Because of the strong association of pembrolizumab exposure with the combination of marked troponin elevation, new left ventricular dysfunction, CK elevation consistent with myositis/rhabdomyolysis, lung findings concerning for pneumonitis, and worsening renal function, concern increased for pembrolizumab-associated myocarditis with multisystem immune toxicity.

Pembrolizumab was permanently discontinued. Systematic corticosteroids were initiated, with oral prednisone 40 mg twice daily. The regimen was continued for 4 days, during which the patient demonstrated clinical improvement. The corticosteroid regimen was subsequently transitioned to prednisone 60 mg once daily for 3 days, and the patient was discharged on this dose with a plan for outpatient oncology follow-up to guide further steroid dosage tapering. Cardiac magnetic resonance imaging and endomyocardial biopsy were not performed. Given the patient's clinical stability, advanced age, comorbid chronic kidney disease, and high clinical suspicion for immune-mediated myocarditis with prompt symptomatic improvement after corticosteroid initiation, the cardiology team recommended deferring further invasive or contrast-based diagnostic testing. The cardiology, oncology, pulmonology, and nephrology teams were involved early in the patient's care.

## Outcome and Follow-Up

After initiation of systemic corticosteroids, the patient experienced gradual improvement in dyspnea, with decreasing oxygen requirements accompanied by a decline in high-sensitivity troponin (from a peak of 6,598 ng/L to 4,192 ng/L within 2 days of treatment initiation) and CK levels (from 4,147 to 1,114 U/L). Further serial troponin measurements were not obtained after the initial decline owing to clinical improvement and stabilization. Renal function improved and returned to baseline creatinine levels without the need for renal replacement therapy. The LBBB persisted during hospitalization, without progression to high-grade atrioventricular block or malignant arrhythmias. At the time of discharge, the patient remained clinically stable, with improving functional status. She was discharged on oral prednisone 60 mg once daily with close outpatient follow-up planned with her treating oncologist to guide further steroid dose tapering. Subsequent outpatient follow-up records were not available for review at the time of manuscript preparation.

## Discussion

ICI-associated myocarditis is uncommon, but it can be a severe life-threatening complication that may occur early, sometimes after 1 or 2 doses of therapy.^2^ The incidence of myocarditis while on single-agent pembrolizumab is thought to be between 0.06% and 1%—extremely rare.^2^ It may also present together with other immune-related toxicities, particularly myositis and pneumonitis.^2^^,^^3^ In older patients with baseline cardiovascular and renal disease, it can be hard to recognize because the symptoms and laboratory results can look like more common problems such as acute coronary syndrome or heart failure.

In this case, the combination of new conduction abnormalities, LBBB, marked troponin elevation, new drop in ejection fraction, simultaneous CK elevation soon after exposure to pembrolizumab, and exclusion of obstructive coronary disease supported the diagnosis of immune-mediated myocarditis. Although the patient was treated with single-agent ICI therapy, her advanced age, chronic kidney disease, and pre-existing cardiovascular disease may have increased susceptibility to severe immune-related toxicity. These comorbidities may help explain the rapid onset and multisystem presentation despite limited exposure to single-agent immunotherapy. The key steps were to simultaneously evaluate for common serious causes such as acute coronary syndrome and pulmonary embolism, while keeping immune toxicity also on the differential. Once immune toxicity was strongly suspected, early initiation of high-dose corticosteroids and multidisciplinary management likely contributed to clinical stabilization and recovery.^4^

This case highlights the need to consider immune-related cardiotoxicity in patients receiving ICIs, even in the adjuvant setting, and the importance of early coordinated multidisciplinary involvement.^4^

## Conclusions

Early multisystem immune-related toxicity, including myocarditis, can occur after limited exposure to pembrolizumab in elderly patients receiving adjuvant treatment for renal cell carcinoma. Rapid exclusion of ischemic disease and early initiation of systemic corticosteroids are essential to improve outcomes.

**Visual Summary:** Timeline of the Case

Time	Events
Day 0	An 84-year-old woman with history of renal cell carcinoma after having undergone left nephrectomy, chronic kidney disease, hypertension, diabetes mellitus, and chronic heart failure presented with worsening shortness of breath for 2 days and inability to lie flat after receiving immune checkpoint inhibitor therapy (pembrolizumab).
Day 0	Initial evaluation revealed markedly elevated high-sensitivity troponin with significant rise, elevated creatinine, and elevated CK. Electrocardiogram showed left bundle branch block without ischemic ST-segment changes. Chest radiograph showed no acute pulmonary edema.
Day 1	Patient was admitted for presumed NSTEMI and treated with heparin infusion, diuretics, beta-blocker therapy, and oxygen supplementation. Cardiology and oncology were consulted.
Days 1-2	Transthoracic echocardiography demonstrated mildly reduced left ventricular systolic function with septal hypokinesis. Coronary angiography showed no obstructive coronary artery disease, making acute coronary syndrome less likely.
Day 2	Continued rise in troponin and CK levels, worsening renal function, and development of mild hypoxemia raised concern for immune checkpoint inhibitor–related myocarditis and myositis. Pembrolizumab was discontinued.
Days 2-3	High-dose systemic corticosteroids were initiated (prednisone 40 mg twice daily). Anticoagulation was discontinued. Close cardiac and pulmonary monitoring continued.
Days 3-5	After steroid initiation, troponin and CK levels began to downtrend, renal function improved and returned to baseline, respiratory symptoms improved, with decrease in oxygen requirements.
Days 5-7	Patient was transitioned to prednisone 60 mg once daily, with clinical improvement. Patient was discharged to home on prednisone 60 mg once daily in stable condition, with close cardiology and oncology follow-up to guide steroid taper.
Discharge/early postdischarge status	At the time of discharge, the patient remained clinically stable with no recurrent dyspnea, no malignant arrythmias, improving functional status, and continued clinical improvement. Outpatient records were not available.

Note: Objective documentation of complete resolution of myocarditis, conduction abnormalities, and pneumonitis was not available; therefore, improvement was described based on clinical and laboratory trends.

CK = creatine kinase; NSTEMI = non–ST-segment elevation myocardial infarction.

## Funding Support and Author Disclosures

The authors have reported that they have no relationships relevant to the contents of this paper to disclose.
